# Effects of different endotracheal tube cuff management on sore throat, hoarseness, and cough after short-term gynecological laparoscopic surgery

**DOI:** 10.1038/s41598-026-47172-8

**Published:** 2026-04-04

**Authors:** Qian Li, Xiaoling Jin, Ailing Tu, Faping Tu

**Affiliations:** 1 Department of Anesthesia, Obstetrics and Gynecology Hospital of Fudan University, Shanghai Key Lab of Reproduction and Development, Shanghai Key Lab of Female Reproductive Endocrine Related Diseases, Shanghai, China; 2 Department of Anesthesiology, CHENGDU SIXTH PEOPLE’S HOSPITAL, Chengdu, China; 3 Department of Anesthesiology, North Sichuan Medical College, Nanchong, China; 4 Department of Clinical Medicine, Grade 2017, North Sichuan Medical College, Nanchong, China

**Keywords:** Gynecological laparoscopic surgery, Cuff management, Sore throat, Hoarseness, Cough, Medical research, Risk factors

## Abstract

Sore throat, hoarseness and cough are common airway complications after extubation. In this study, we compared the effects of three different management for endotracheal tube cuff on postprocedural airway complications. 180 patients undergoing short-term gynecological laparoscopic surgery were randomly divided into three groups (*n* = 60, per group): digital palpation method group (group A), minimal occlusive volume method group (group B) and pressure control method group (group C). The primary objective was incidence and severity of POST. Secondary outcomes was incidence and severity of postoperative hoarseness, cough. We hypothesize that measuring and controlling ETTc may reduce the incidence of postoperative pharyngeal complications in those patients. The incidence and severity of sore throat, hoarseness, and cough were measured at 1, 6, and 24 h after tracheal extubation. The incidence and severity of sore throat in group B and C were significantly lower than those in group A at 1 h and 6 h after extubation (47.4% vs. 25.5% vs.17.5%, *P* = 0.016, 0.003; 54.2% vs. 29.1% vs.19.6%, *P* = 0.003, 0.000; severity scores: *P* = 0.01, 0.003; *P* < 0.01, = 0.000), but no significant differences between the three groups were seen at 24 h after surgery. The incidence of and severity of sore throat was similar between the group B and group C. There were no significant differences in the incidence and severity of hoarseness and cough at 1, 6 and 24 h after extubation among the three groups (*P* > 0.05). The severity of tracheal mucous injury in group A was more severe than the group B and group C (*P* = 0.013, < 0.001). Both the manometry method and the minimal occlusive volume method may reduce the incidence and severity of sore throat in the short term after extubation in short-term laparoscopic gynecologic surgery.

**Supplementary Information:**

The online version contains supplementary material available at 10.1038/s41598-026-47172-8.

## Introduction

Postoperative sore throat (POST), cough, and hoarseness are common airway complications after tracheal intubation, with an incidence varying from 14.4–62%^1–3^. The physiopathology of post-intubation airway symptoms is not completely clarified, but mucosal damage due to high cuff pressure is thought to be an essential causative factor^4–6^. The control of endotracheal tube (ETT) cuff pressure during surgery is an integral aspect of anesthesia. If the ETT cuff (ETTc) pressure is greater than 30 cmH_2_O, the airway mucosa could be damaged, and some airway complications would occur^4,7^. It is recommended to maintain cuff pressure in the range of 25–30 cmH_2_O (18–22 mmHg) after inflation, lower than the capillary perfusion pressure of the tracheal mucosa^6,8–10^.

Laparoscopic gynecological surgery has become popular over the last decade, decreasing postoperative pain, shortening hospital stays, and reducing medical costs^11^. However, the CO_2_ pneumoperitoneum and Trendelenburg position during laparoscopy may increase ETTc pressure and lead to discomfort and complications postoperatively^12,13^. Previous studies have shown that female patients are at a greater risk for POST after ETT^14,15^.

Multiple techniques exist for the inflation of the endotracheal cuff, such as the manual palpation of the pilot balloon, minimum occlusive volume (MOV) technique and inflation to precise pressure and continuous monitoring. Manual palpation of the pilot balloon, while convenient, frequently results in intracuff pressures well above the recommended threshold of 30 cmH_2_O, and there is no good correlation between this and the cuff pressure measured by the pressure gauge^16,17^. The minimum occlusive volume (MOV) tends to produce pressures that are marginally insufficient, often requiring a further addition of around 0.5 mL of air or increasing the cuff pressure by around 4 cmH_2_O to ensure a complete seal^18^. In contrast, continuous pressure monitoring allows for precise, real-time adjustment to maintain cuff pressure within the recommended range.

While multiple randomized controlled trials have established the necessity of continuous cuff pressure monitoring during long-duration surgeries^9^^19^, this practice is often neglected in procedures lasting only a few hours. In such cases, clinicians commonly determine the pressure by pilot balloon palpation according to their experience. With the growing emphasis on comfort-oriented medical care, even minor complications such as postoperative sore throat, hoarseness, or cough have gained increasing significance as they directly impact patient satisfaction and recovery quality^3^. This raises an important clinical question: Does the management of tracheal tube cuff pressure add to the workload of the physician or provide clinical benefits to the patient?

At present, no studies have investigated the differential effects of different tracheal tube cuff management strategies on postoperative pharyngeal complications in patients undergoing short-duration laparoscopic gynecological surgery. Therefore, we hypothesize that measuring and controlling ETTc in patients undergoing short-during laparoscopic gynecological surgery may also reduce the incidence of postoperative pharyngeal complications. The primary objective of this study was to compare the effect of digital palpation method, minimal occlusive volume method and pressure control method on the incidence and the severity of POST.The incidence and severity of postoperative hoarseness, cough as secondary outcomes.

## Results

**Fig. 1:**
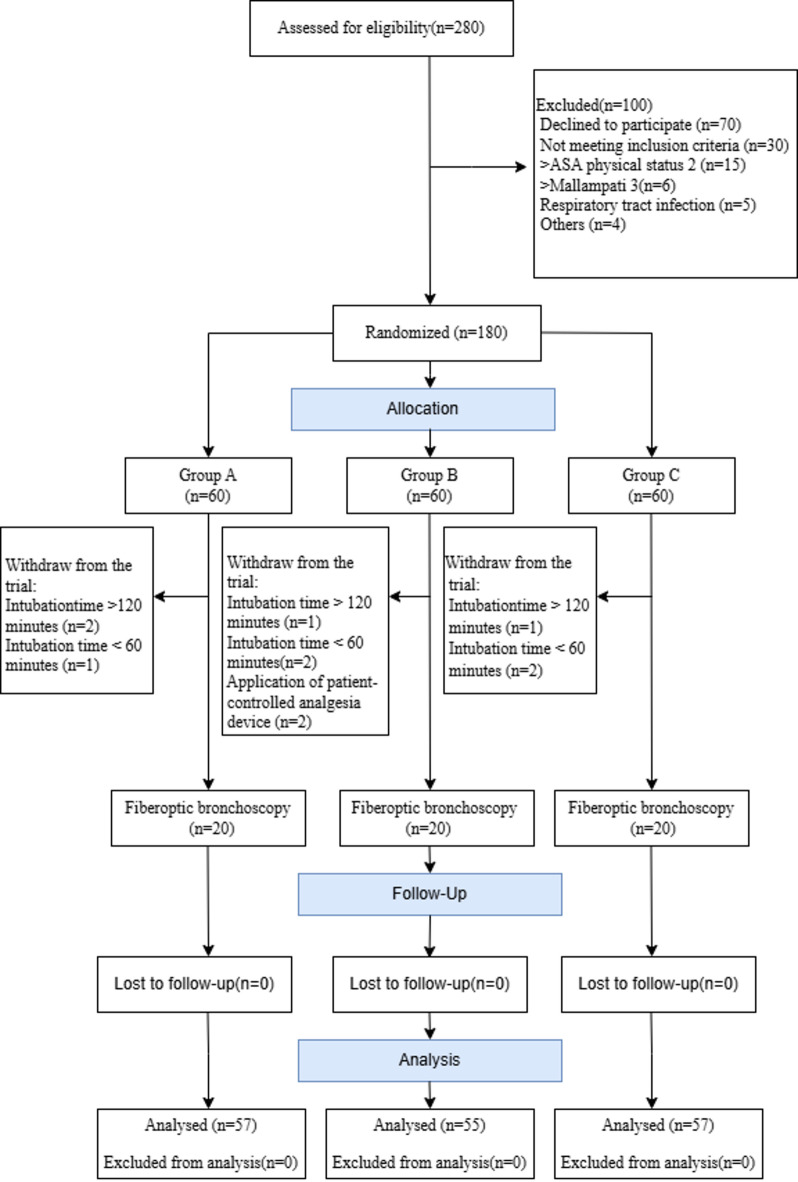
Flow chart of patients enrolled in the study.

One hundred eighty patients met the inclusion criteria and consented to participate in the study. 3, 5, and 3 patients withdrew from the digital-palpation method group (group A), minimal-occlusive-volume method group (group B), and pressure-control method group (group C), respectively, because 4 patients had an intubation time of more than 120 min, 5 patients had a intubation time of less than 60 min and 2 patients used postoperative analgesia pumps. Therefore, 169 patients were enrolled in this study. None of the patients were lost to follow-up. A flow chart of the patients involved in the study is shown in Fig. 1.

The characteristics of the patients and Intraoperative Variables were comparable across the groups (Table 1). There were no differences in age, BMI, ASA classification, inclination of the operating table among the three groups. The Intraoperative Variables such as operation duration, duration of endotracheal intubation and dosage of anesthetic showed no significant difference within the groups.

**Table 1:** Patient characteristics and intraoperative variables.

	Group A	Group B	Group C	*P* value
	(*n* = 57)	(*n* = 55)	(*n* = 57)	
Age (years)	38.3 ± 11.1	38.8 ± 10.4	39.9 ± 10.5	0.742
Preoperative haemoglobin (g/L)	126.6 ± 16.9	122.8 ± 18.8	120.0 ± 17.6	0.178
BMI (kg m^−2^)	21.6 ± 2.5	22.3 ± 2.5	22.5 ± 3.0	0.206
Inclination of the operating table (°)	21.7 ± 3.3	21.6 ± 3.5	21.2 ± 3.0	0.71
ASA status Ⅰ/Ⅱ (%)	26/24	24/28	29/21	1.424
Duration of tracheal intubation (min)	125.0 ± 29.8	124.1 ± 28.5	120.7 ± 24.3	0.715
Duration of the operation (min)	104.1 ± 28.1	102.8 ± 25.5	97.4 ± 22.8	0.387
Duration of pneumoperitoneum (min)	94.4 ± 27.3	91.0 ± 24.9	88.5 ± 21.8	0.495
Mean propofol dose (mg)	122.4 ± 35.7	129.6 ± 42.5	128 ± 39.8	0.699
Mean sufentanil dose (μg)	33.3 ± 4.2	32.5 ± 3.9	31.7 ± 3.8	0.847
Mean Rocuronium dose (mg)	38.5 ± 6.0	39.1 ± 8.6	39.9 ± 8.5	0.786
Infusion volume (ml)	1893.0 ± 500.0	1866.3 ± 443.1	1869.0 ± 359.0	0.873
Blood loss (ml)	50.0 (20.0-100.0)	50.0 (20.0-100.0)	50.0 (20.0-100.0)	0.672

Data are presented as the mean (SD), median (range) or numbers (proportion, %).

ASA, American Society of Anaesthesiologists; group A, digital-palpation method group; group B, minimal-occlusive-volume method group; group C, pressure-control method group; BMI, body mass index. Differences in age, Preoperative haemoglobin, BMI, Inclination of the operating table, Duration of tracheal intubation, Duration of the operation, Duration of pneumoperitoneum, Mean Propofol dose, Mean Sufentanil dose, Mean Rocuronium dose, Infusion volumebetween groups were analyzed using one-way ANOVA, and in ASA status and Blood loss between groups were analysed using the χ^2^ test. No significant difference was found between the groups.

**Fig. 2:**
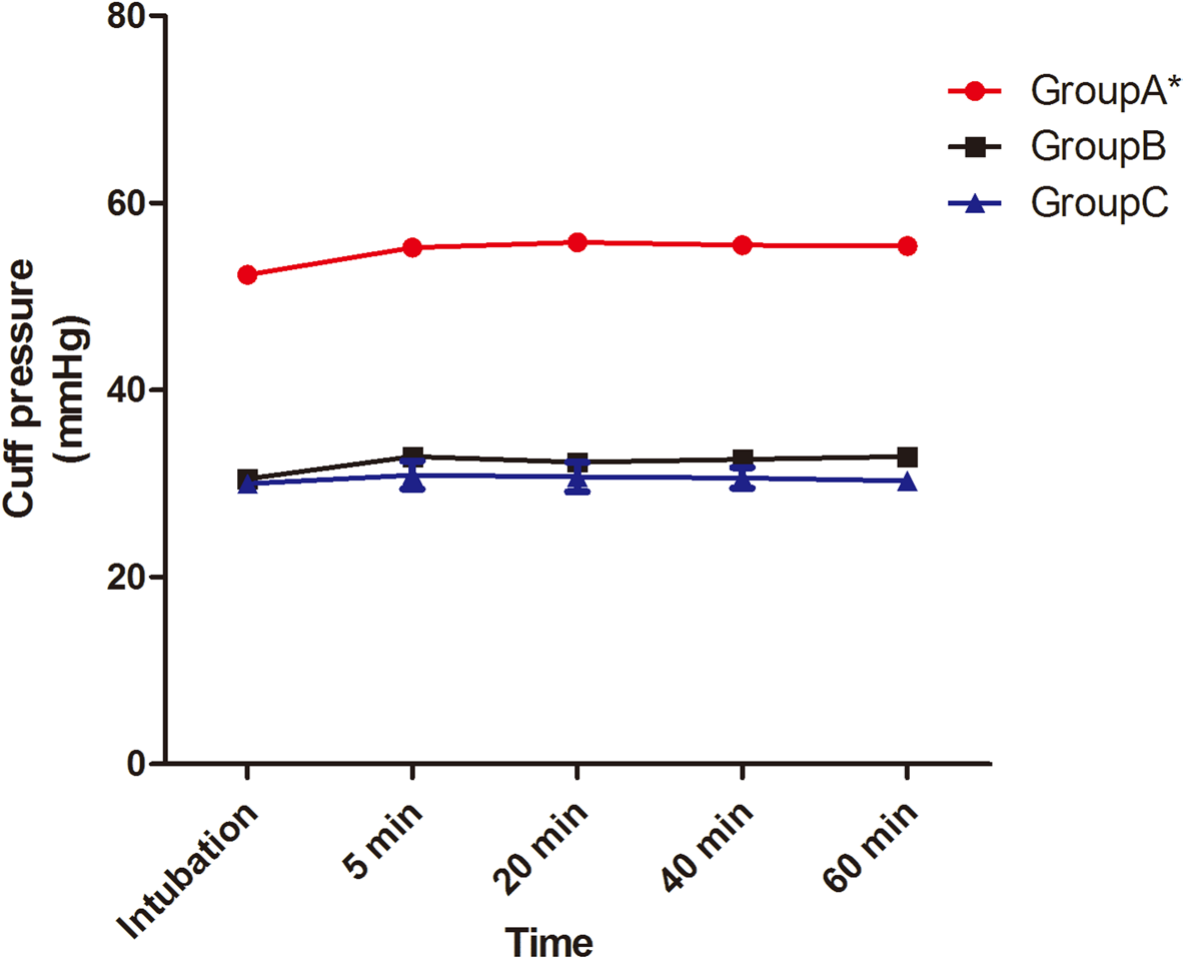
The cuff pressures among the groups. * *P* < 0.05, significantly different from Group B and Group C. Group A indicates the digital-palpation method group; group B, the minimum-occlusive-volume method group; and group C, the pressure-control method group.

The endotracheal cuff pressures in the digital-palpation method group were significantly higher than those in the other groups at all time points studied (*P*<0.05) (Fig. 2).

**Fig. 3:**
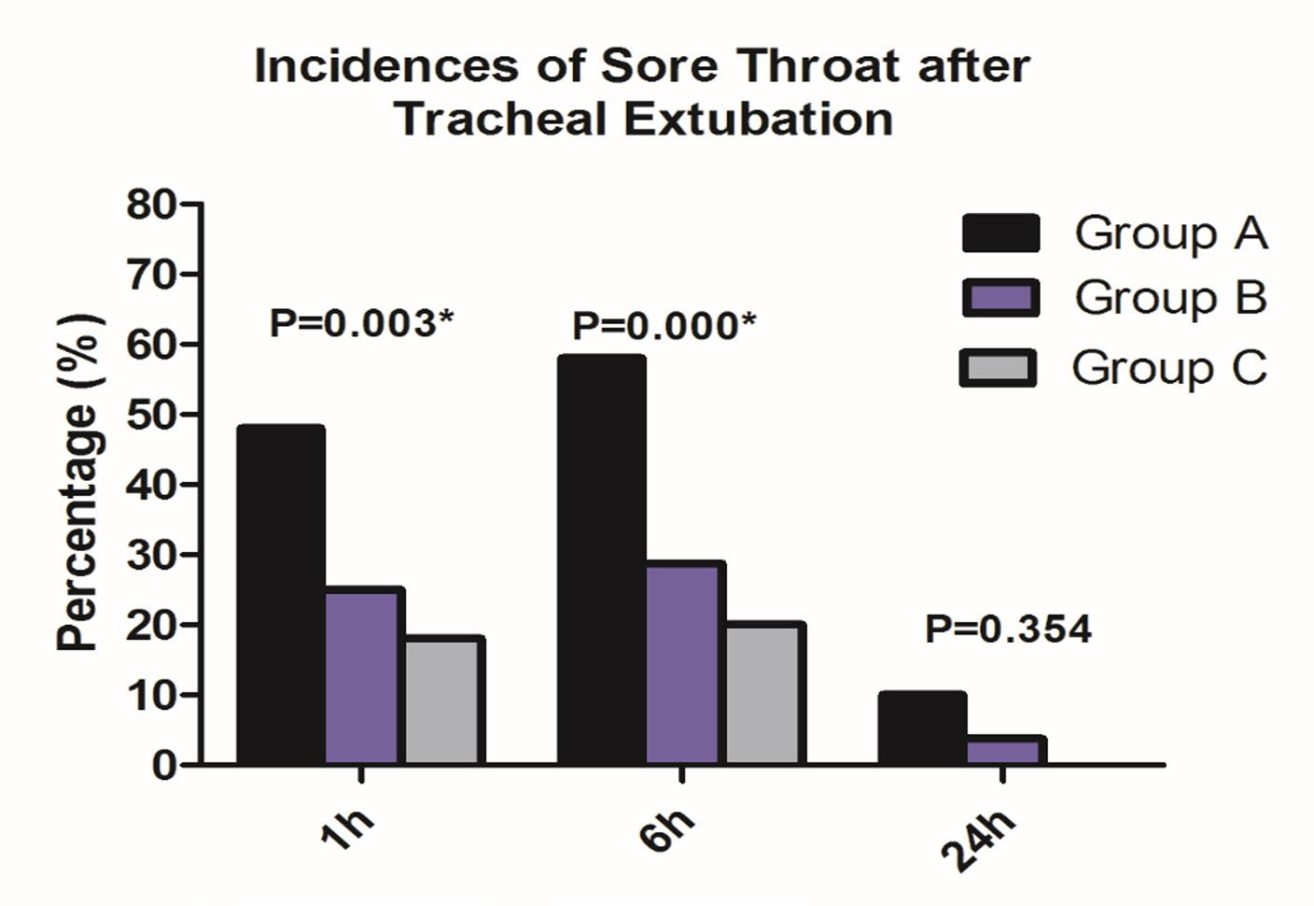
Incidences of sore throat among the groups after tracheal extubation. The incidence of the Sore Throat at 1, 6, and 24 h after extubation were 47.4% (27/57; 95% confidence interval [CI], 0.474 [0.340–0.607]),54.2% (32/57; 95% confidence interval [CI], 0.542 [0.411–0.673]) and 10.5.% (6/57; 95% confidence interval [CI], 0.105 [0.230–0.187]) in the groupA.The incidence of the Sore Throat at 1, 6, and 24 h after extubation were25.5% (14/55; 95% confidence interval [CI], 0.255 [0.136–0.373]), 29.1% (16/55; 95% CI, 0.291 [0.167–0.415]) and 3.6% (2/55; 95% CI, 0.036 [0.015–0.087]) in the group B. The incidence of the Sore Throat at 1, 6, and 24 h after extubationwere 17.5% (10/57; 95% confidence interval [CI], 0.175 [0.074–0.277]),19.6% (11/57; 95% confidence interval [CI], 0.196 [0.089–0.304]) and 0% in group C. * *P* < 0.0167, significantly different from Group B and Group C at 1 h and at 6 h. Group A indicates the digital-palpation method group; group B, the minimum-occlusive-volume method group; group C, the pressure-control method group; 1 h: 1 h after extubation; 6 h: 6 h after extubation; 24 h: 24 h after extubation.

The incidence of POST between the 3 groups at time intervals of 1, 6, and 24 h is shown in Fig. 3. The incidence of the Sore Throat at 1, 6, and 24 h after extubation were 47.4% (27/57; 95% confidence interval [CI], 0.474 [0.340–0.607]), 54.2% (32/57; 95% confidence interval [CI], 0.542 [0.411–0.673]) and 10.5.% (6/57; 95% confidence interval [CI], 0.105 [0.230–0.187]) in the group A.The incidence of the Sore Throat at 1, 6, and 24 h after extubation were25.5% (14/55; 95% confidence interval [CI], 0.255 [0.136–0.373]), 29.1% (16/55; 95% CI, 0.291 [0.167–0.415]) and 3.6% (2/55; 95% CI, 0.036 [0.015–0.087]) in the group B. The incidence of the Sore Throat at 1, 6, and 24 h after extubationwere 17.5% (10/57; 95% confidence interval [CI], 0.175 [0.074–0.277]),19.6% (11/57; 95% confidence interval [CI], 0.196 [0.089–0.304]) and 0% in group C. There was a significantly higher incidence of POST in the digital- palpation method group (47.4%) than in the minimal-occlusive-volume method group (25.5%) and the pressure-control method group (17.5%) (*P* = 0.016 and 0.003) at a time interval of 1 h. At a time interval of 6 h, the POST incidence of the digital-palpation method group was 54.2% higher than that of the minimal-occlusive-volume method group (29.1%) and the pressure-control method group (19.6%) (*P* = 0.003 and 0.000). Twenty-four hours postoperatively, 10.5% of the digital-palpation method group, 3.6% of the minimum-occlusive-volume method group and 0% of the pressure-control method group reported POST (*P* = 0.354), which was not statistically significant (Fig. 3).

The intensity of sore throat decreased over time postoperatively, with the worst pain at 1 h after surgery and the least at 24 h after surgery. Compared with the minimum-occlusive-volume method group and the pressure-control method group, the digital-palpation method group had significantly increased severity of POST at 1 and 6 h after extubation (*p* = 0.01,<0.01 and *p* = 0.003, 0.000). In the pressure-control method group, no patient experienced moderate POST (Table 2).

**Table 2:** The Tracheal mucous injury and The Sore Throat, Hoarseness, and Cough severity score at different time in the three groups.

	Time points	Severity score	Group A (*n* = 57)	Group B (*n* = 55)	Group C (*n* = 57)	P-values			
						Among three groups	Between AAnd B groups	Between AAnd C groups	Between BAnd C groups
Sore Throat	1 h after extubation	0	29 (50.9)	41 (74.9)	47 (82.5)	< 0.01	0.01*	< 0.01*	0.308
		1	23 (40.4)	13 (23.6)	10 (17.5)				
		2	5 (8.8)	2 (3.6)	0 (0.0)				
		3	0 (0.0)	0 (0.0)	0 (0.0)				
	6 h after extubation	0	24 (42.1)	39 (70.9)	46 (80.7)	0.000	0.003*	0.000*	0.299
		1	31 (54.3)	15 (27.3)	11 (19.2)				
		2	2 (3.5)	1 (1.8)	0 (0.0)				
		3	0 (0.0)	0 (0.0)	0 (0.0)				
	24 h after extubation	0	51 (89.5)	53 (96.4)	57 (100.0)	0.354			
		1	6 (10.5)	2 (3.6)	0 (0.0)				
		2	0 (0.0)	0 (0.0)	0 (0.0)				
		3	090.0)	0 (0.0)	0 (0.0)				
Hoarseness	1 h after extubation	0	35 (61.4)	34 (61.8)	39 (68.4)	0.753			
		1	15 (26.3)	18 (32.7)	18 (31.6)				
		2	6 (10.5)	3 (5.5)	0 (0.0)				
		3	1 (1.8)	0 (0.0)	0 (0.0)				
	6 h after extubation	0	41 (71.9)	35 (63.6)	44 (77.2)	0.266			
		1	15 (26.3)	18 (32.7)	13 (22.8)				
		2	0 (0.0)	2 (3.6)	0 (0.0)				
		3	1 (1.8)	0 (0.0)	0 (0.0)				
	24 h after extubation	0	56 (98.2)	53 (96.3)	56 (98.2)	0.806			
		1	0 (0.0)	1 (1.8)	1 (1.7)				
		2	1 (1.8)	1 (1.8)	0 (0.0)				
		3	0 (0.0)	0 (0.0)	0 (0.0)				
Cough	1 h after extubation	0	46 (80.7)	47 (85.5)	42 (73.7)	0.414			
		1	11 (19.3)	8 (14.5)	15 (26.3)				
		2	0 (0.0)	0 (0.0)	0 (0.0)				
		3	0 (0.0)	0 (0.0)	0 (0.0)				
	6 h after extubation	0	51 (89.5)	48 (87.2)	52 (91.2)	0.662			
		1	6 (10.5)	7 (12.7)	5 (17.5)				
		2	0 (0.0)	0 (0.0)	0 (0.0)				
		3	0 (0.0)	0 (0.0)	0 (0.0)				
	24 h after extubation	0	56 (98.2)	55 (100.0)	54 (94.7)	0.159			
		1	1 (1.8)	0 (0.0)	3 (5.3)				
		2	0 (0.0)	0 (0.0)	0 (0.0)				
		3	0 (0.0)	0 (0.0)	0 (0.0)				

Values are number of patients. group A, digital-palpation method group; group B, minimal-occlusive-volume method group; group C, pressure-control method group, Differences in the Sore Throat, Hoarseness, and Cough severity score at different time between groups were analysed using χ^2^ or Fisher’s exact test. Bonferroni correction test was used for further statistical comparison between groups. *P < 0.0167 vs.group A.

**Fig. 4:**
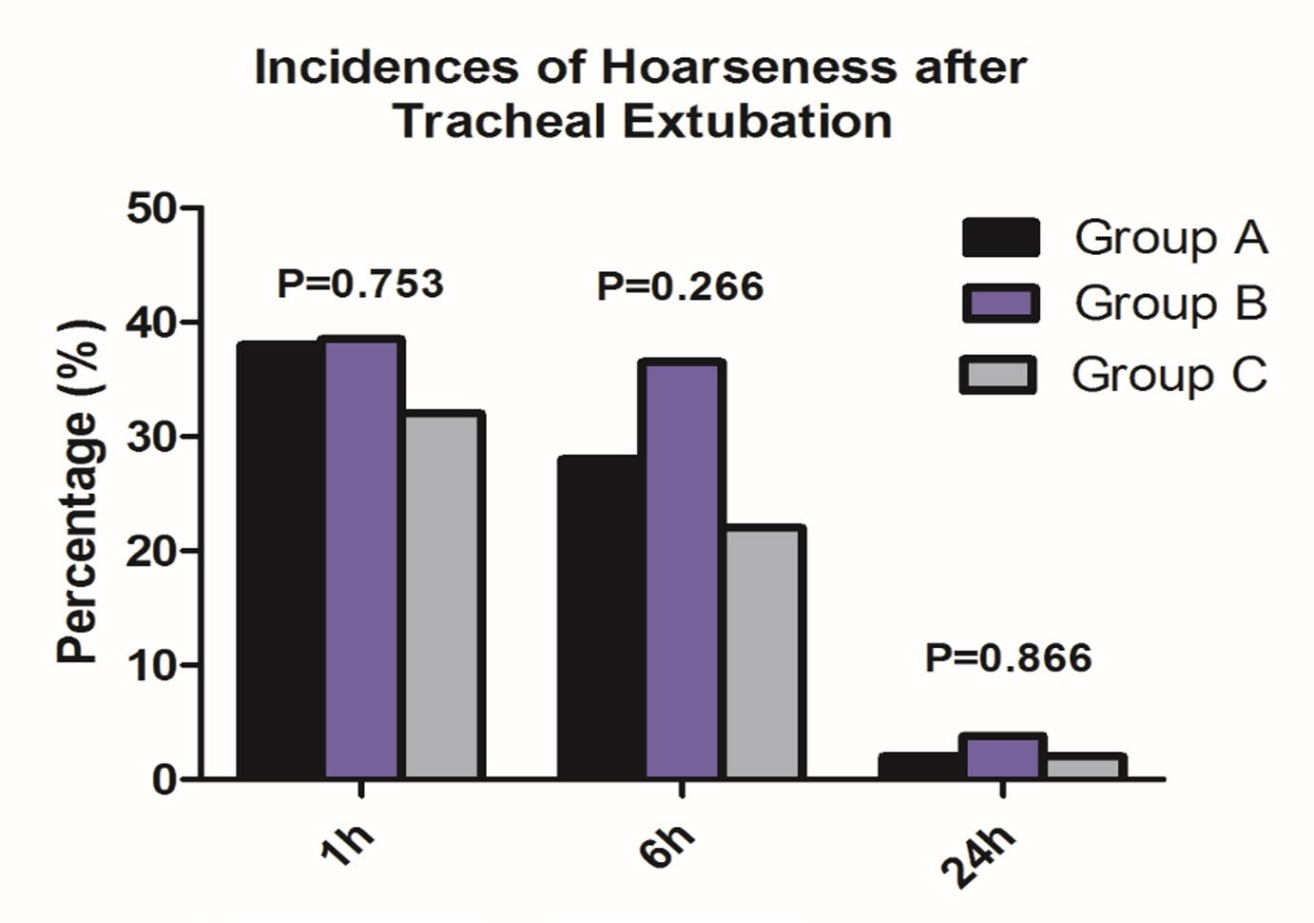
Incidences of Hoarseness among the groups after tracheal extubation. Group A indicates the digital-palpation method group; group B, the minimum-occlusive-volume method group; group C, the pressure-control method group; 1 h, 1 h after extubation; 6 h, 6 h after extubation; 24 h, 24 h after extubation.

**Fig. 5:**
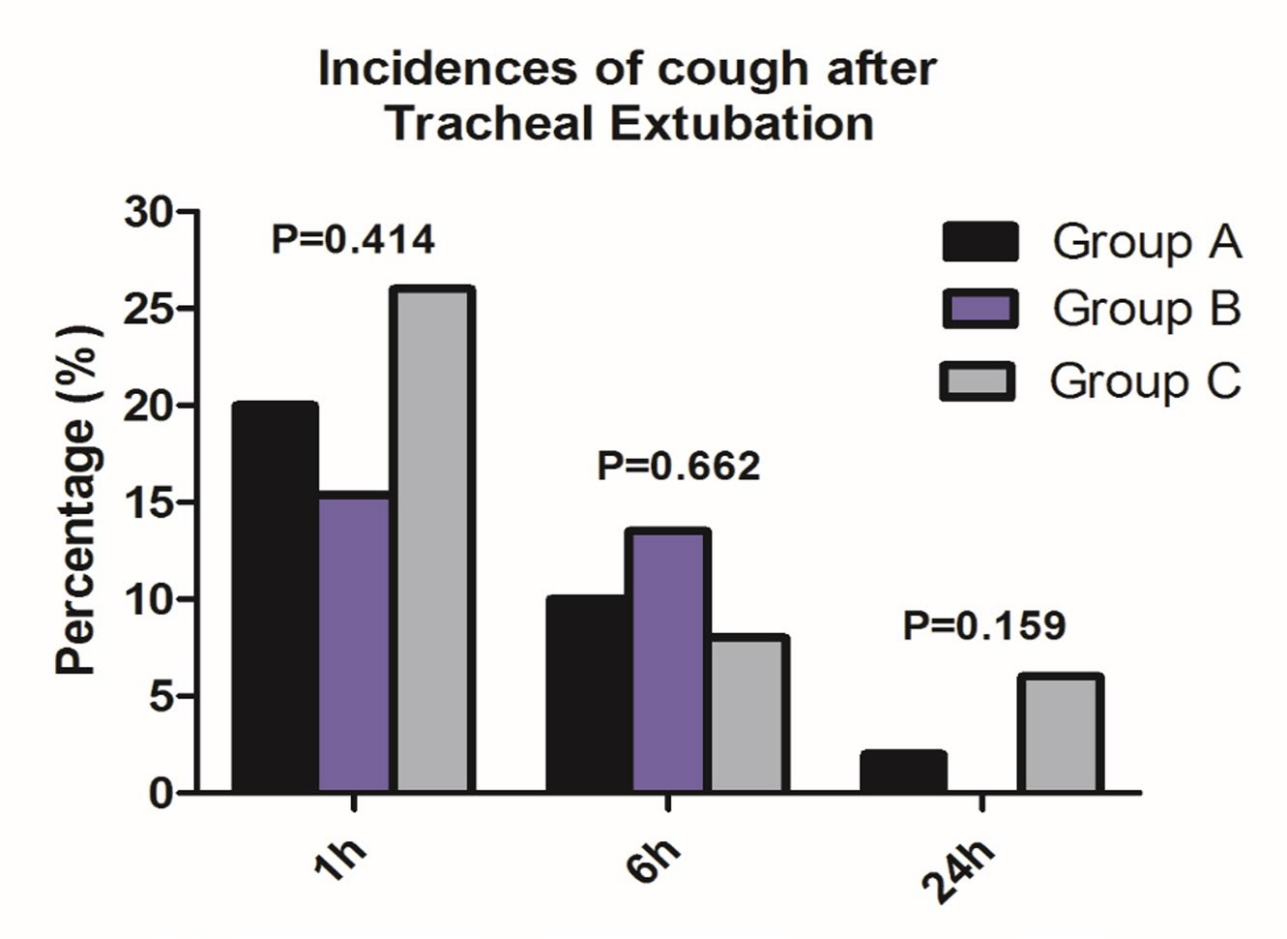
Incidences of cough among the groups after tracheal extubation. Group A indicates the digital-palpation method group; group B, the minimum-occlusive-volume method group; group C, the pressure-control method group; 1 h, 1 h after extubation; 6 h, 6 h after extubation; 24 h, 24 h after extubation.

The incidence of hoarseness and the severity of hoarseness were not significantly different among the three groups, but in the digital-palpation method group, 1 patient experienced severe hoarseness (Table 2; Fig. 4).The incidence of cough and the severity of cough were not significantly different among the three groups (Table 2; Fig. 5).

**Fig. 6:**
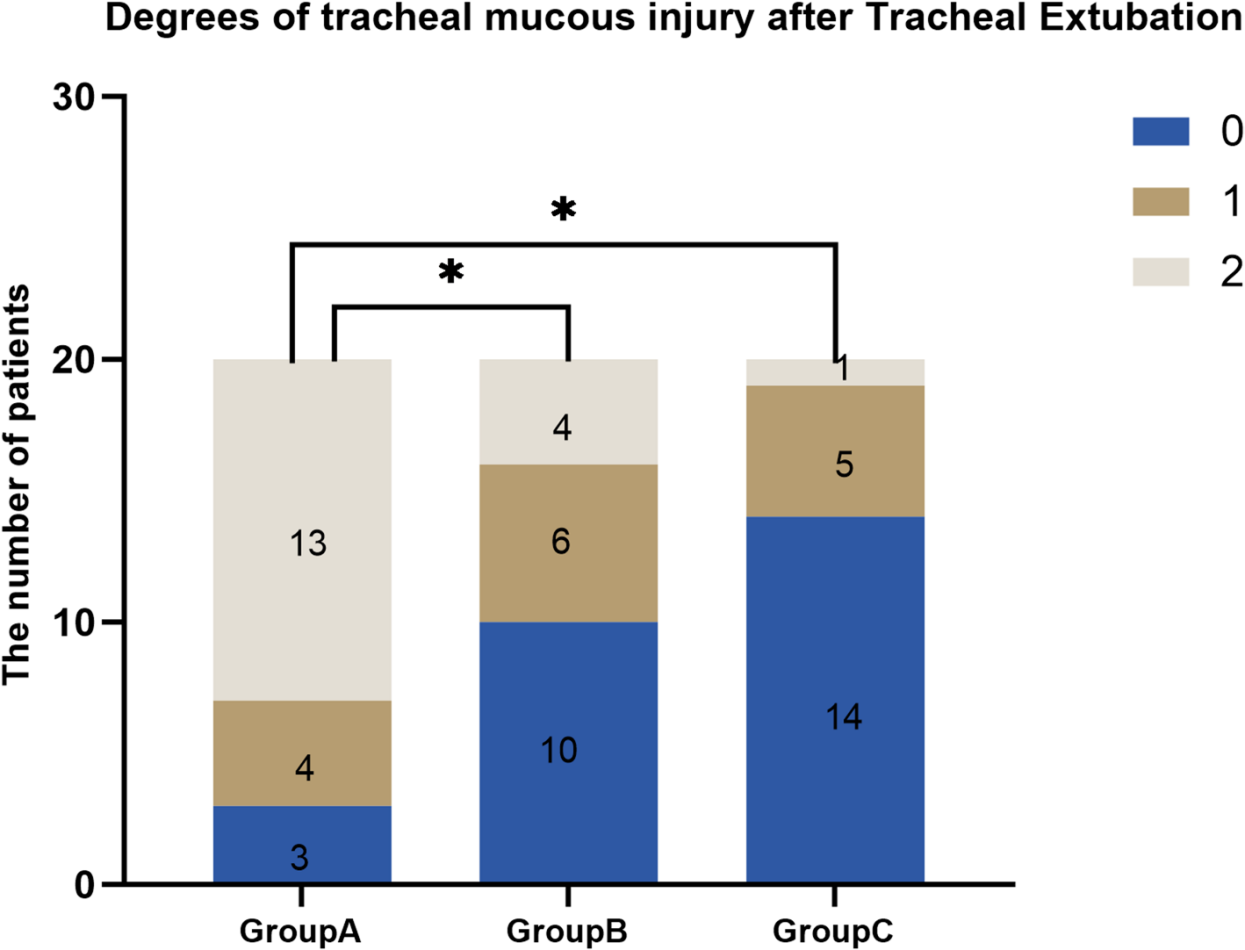
The degrees of tracheal mucous injury after tracheal extubation among the groups. * *P* < 0.0167, significantly different from Group B and Group C. Group A Indicates the digital-palpation method group; group B, the minimum-occlusive-volume method group; group C, the pressure-control method group.

The severity of tracheal mucous injury evaluated by bronchofiberscope at the end of surgery in the digital-palpation method group was more severe than the minimal-occlusive-volume method group and the pressure-control method group (*P =* 0.013, < 0.001).r No statistically significant difference was found between the minimal-occlusive-volume method group and the pressure-control method group, and no severe tracheal mucous injury was found in any of the three groups (Table 3; Fig. 6).

**Table 3:** Tracheal mucous injury in the three groups.

	Group A (*n* = 20)	Group B (*n* = 20)	Group C (*n* = 20)	*P*-values			
				Among three groups	Between A And B groups	Between A And C groups	Between B And C groups
Tracheal mucous injury (score)	2.0 (1.0–2.0)	0.5 (0–1.0)	0 (0–1.0)	< 0.01	0.013*	< 0.001*	0.207

The values are expressed as median (25-75th percentiles).group A, digital-palpation method group; group B, minimal-occlusive-volume method group; group C, pressure-control method group Difference in tracheal mucous injury using the Kruskal-Wallis test. Bonferroni correction test was used for further statistical comparison between groups. *P < 0.0167 vs.group A.

## Discussion

In this prospective randomized controlled trial, we found measuring and controlling ETTc substantially reduced the incidence and severity of POST in patients undergoing short-during laparoscopic gynecological surgery after general endotracheal anesthesia. Additionally, bronchoscopy verified that the measuring and controlling ETTc had less mucosal injury. However, the incidence of hoarseness and cough and their severity were comparable. Our findings will not only contribute to the existing body of knowledge regarding the impact of endotracheal tube cuff pressure on airway complications after extubation but also provide clear guidelines for clinical practice, optimizing patient comfort and safety.

Although considered a minor complaint after anesthesia, POST is an important consideration for patients. POST is a complication that might occur after ETT insertion in patients undergoing general anesthesia and is related to mucosal damage and mechanical damage due to friction and pressure between the device and ETTc pressure on the pharyngeal mucosa during insertion and anesthesia, which leads to inflammation and triggers several postoperative symptoms, such as sore throat, dysphagia, and dysphonia^3,20^. A continuous lateral wall pressure above 30 cmH_2_O (22 mm Hg) can impair mucosal capillary blood flow. This flow is totally blocked when the lateral wall pressure reaches 50 cmH_2_O (37 mm Hg). This is the most important cause of mucosal damage and can be avoided by careful attention to the inflation technique and continuous monitoring of the intracuff pressure^6^. Liu et al. reported that proper control of ETTc pressure by a manometer, even in procedures lasting only a few hours, helps to reduce postprocedural endotracheal intubation-related complications^21^.

It is important to maintain an adequate ETTc pressure not only to avoid ventilatory leaks during mechanical ventilation but also to prevent aspiration, especially for patients in the head-down position^22^. During laparoscopic surgery, the ETTc pressure increases with abdominal insufflation, and the patient position, changes, especially the head-down position^13,23^. Unfortunately, most clinicians give little attention to the inflation pressure of the ETTc and simply determine the pressure by pilot balloon palpation according to their experience. In fact, several postoperative complications, such as cough, sore throat, hoarseness and blood-streaked expectorations, are associated with high ETTc pressure^21,24^. In our study, the ETTc pressure of the endotracheal tube progressively increased during pneumoperitoneum in the three groups, and the ETTc pressure increased more in group A, from 52 cmH_2_O to 56 cmH_2_O. It can be seen that the ETTc pressure range fluctuates greatly when the ETTc is managed by the sensory method and is much higher than the ideal ETTc pressure (20–34 cmH_2_O), indicating that the patient’s tracheal mucosa will be subjected to higher ETTc pressures using the pilot balloon palpation method, and airway complications after extubation may increase. The 54.2% incidence of POST in group A suggests that the potential for injury is considerable when cuff management is overlooked, irrespective of the short surgical duration^21^.

The stethoscope-guided method of tracheal tube ETTc inflation involves applying the least amount of air injection after intubation to seal the capsule and prevent it from leaking, in order to avoid excessive or insufficient inflation to the ETTc; thus, the pressure of the ETTc will not exhibit obvious fluctuation. Our results showed that the ETTc pressure in group B was 30.5 ± 8.7 cmH_2_O at T_0_, which was significantly lower than the pressure of 52.6 ± 18.0 cmH_2_O in group A, indicating that the management of the ETTc by the minimum-closed-volume method would not cause excessive capsule pressure. However, the ETTc pressure in group B was higher than 20 cmH_2_O, as reported by Kumar et al.^25^. This difference may be explained by use of the method of auscultation to confirm air leakage sounds or the pressure of manually controlled ventilation.

Several interventions have been attempted to prevent or reduce airway complications after extubation, and controlling pressure is one of the main methods. Lomholt et al. recommended that an ETTc pressure of 25 cmH_2_O should be selected as the safe minimum ETTc pressure to prevent aspiration and leaks past the ETTc^26^. At present, the recommended ETTc pressure is 25 to 30 cmH_2_O^10^. In this study, we continuously monitored the pressure of the ETTc and controlled it at 30 cmH_2_O because the patient experienced air leakage when controlling the pressure of the ETTc at 26 cmH_2_O in the preliminary study. The ETTc pressure after adjustment of group C was 30 cmH_2_O, and the incidences of sore throat at 1 h and 6 h were 17.5% and 19.6%, respectively, which were significantly lower than the incidences of 47.4% and 54.2% in group A. This finding indicates that routine monitoring and controlling of the ETTc pressure after endotracheal intubation even during brief laparoscopic gynecological surgical procedures is helpful in reducing postoperative intubation-related respiratory complications.

The causes of cough, hoarseness, and sore throat after laryngoscopy and endotracheal intubation are attributable to local irritation, inflammation, and oedema in certain parts of the pharynx, larynx, vocal cords, or trachea, and the mechanism remains to be further studied. We found that the incidence and severity of POST, as well as the degree of mucosal injury were significantly high in group A. However, cough and hoarseness were similar for the three groups. These findings were compatible with those of Wang et al.^9^, who found that ETTc pressure elevation led to a higher incidence of POST and similar hoarseness and cough in patients undergoing gynecological laparoscopic surgery. This was also in line with previous results^4,27^ and suggests that these symptoms are not associated with the cuff pressure. Hoarseness results from oedema of the vocal cords from endotracheal intubation, mechanical contact, and abrasion of the tube in the glottal area^21^. Therefore, the application of cricoid pressure during laryngoscopy and the avoidance of forcible intubation had been shown to reduce the incidence of hoarseness^15^. And the ETT cuff is positioned below the glottis and does not directly contact the vocal cords, it is plausible that hoarseness is unrelated to cuff pressure. Similarly, cough in the postoperative period can be triggered by multiple factors including **s**ecretions, blood in the airway, or residual anesthetic effects, which are not directly related to cuff pressure^28^.However, some previous studies also reported that hoarseness was related to increased ETTc pressure^4^.The actual reason for this needs to be determined significantly.

This study also had several potential limitations. First, although we were able to test for primary and secondary outcomes, the sample size was small. The ETTc pressure in group B was higher than that in group C, and the incidence and severity of POST after extubation in group B were also higher than those in group C, but there was no significant difference between the two groups, which may be related to the small sample size in this study^29^. Second, these patients were followed up for only 24 h without a longer follow-up plan to observe their full recovery time and long-term complications. Third, there would be individual differences in sore throat, hoarseness, and cough and the experience of anesthesiologists may also affect the incidence and severity of postoperative airway complications. Furthermore, because not everyone had received a fibreoptic examination to confirm the severity of harm in the postoperative period. The attending anesthesiologists could not be blinded due to the nature of the interventions. Therefore, there are opportunities for bias. Fourth, although coughing during extubation may affect POST^27^, it could not be completely avoided because the patients were extubated while awake. Furthermore, the single-center design and narrow patient selection (short-term gynecological laparoscopic surgeries) may limit the broader applicability of our findings to broader general anesthesia populations.

In conclusion, ETTc pressure estimated by clinical judgement is often much higher than the optimal values to prevent tracheal injury. Compared to routine palpation, both the manometric method and the minimal-occlusive-volume method may decrease the incidence and severity of sore throat in the short term after extubation in laparoscopic gynecologic surgery. Continuous intraoperative measurement of the endotracheal ETTc pressure is a simple and inexpensive procedure and may be considered for patients receiving short-duration laparoscopic gynecologic surgery.

## Methods

### Study design and study population

This prospective, randomized, controlled study was approved by the institutional review board of the Affiliated Hospital of North Sichuan Medical College (2016-001). Prior to enrolling the first participant, the trial was prospectively registered at Chinese Clinical Trial Registry with the registration number ChiCTR-INR-17,010,398 (date of registration 11/01/2017).Written informed consent was obtained from all participants. The study was performed from January 20, 2017, to March 1, 2018, at the Affiliated Hospital of North Sichuan Medical College. The study adhered to the CONSORT guidelines for reporting clinical trials, and all procedures were performed in compliance with the relevant guidelines.

One hundred eighty patients aged between 18 and 60 years with American Society of Anesthesiologists (ASA) physical status I-II requiring elective laparoscopic gynecological surgery that was expected to be no more than 2 h under general anesthesia with endotracheal intubation were prospectively investigated.

The exclusion criteria consisted of a history of recent respiratory tract infection, previous sore throat, hoarseness and cough, cigarette smoking, prior medication with analgesics or corticosteroids, history of heart or lung disease, previous neck surgery, and Mallampati score 3 or 4 .The withdrawal criteria were having tracheal intubation more than twice or failure, oropharyngeal injury during tracheal intubation, a change in surgical plan, allergy during the study, surgery time < 60 min or > 120 min, use of a patient-controlled analgesia, incomplete data collection and voluntary withdrawal of participants.

### Anaesthesia methods

All patients fasted for 8 h before surgery and did not receive premedication. Peripheral venous access was secured with an 18-gauge cannula, and Ringer’s solution was infused and continued at a rate of 5 ml/kg/h during surgery. After preoxygenation for 3 min, anesthesia was induced with midazolam 0.04 mg/kg, propofol 1–2 mg/kg, sufentanil 0.4 ug/kg, neuromuscular block was achieved with rocuronium 0.6 mg/kg. Visual laryngoscopy was used to guide the placement of a 6.5 mm steel wire-reinforced endotracheal tube by an anesthesiologist with at least five years of clinical experience. The cuff was inflated with room-temperature air.

After endotracheal intubation, mechanical ventilation was provided by connecting the tube to the anesthesia machine (Fabius-Plus, Drager, Germany). During surgery, anaesthesia was maintained with sevoflurane, remifentanil and rocuronium. Sevoflurane was adjusted to maintain a bispectral index of 40 to 60 during surgery. To avoid significant fluctuations in cuff pressure during the operation, the use of nitrous oxide was not allowed during the study period. Neuromuscular blockade was monitored, and once the patients conformed to the indications of extubation (TOF ≥ 0.9, EtCO2 < 45 mmHg on spontaneous respiration, and ability to follow voice command), the endotracheal tube cuff was deflated by the same anesthesiologist, and then the tracheal tube was slowly and gently removed.

Electrocardiography, noninvasive arterial blood pressure, and peripheral oxygen saturation were measured during anesthesia. End-tidal PCO_2_ was detected after endotracheal intubation and adjusted to 35 to 45 mm Hg during the operation.

### Evaluation of tracheal mucous injury using a bronchofiberscope

At the end of the surgery, we utilized a fiberoptic bronchoscope (STERRAD 2227552,Olympus, Japan) to examine the tracheal mucosa. Only twenty patients were selected randomly from each group to undergo fiberoptic bronchoscopy because the distal tip of the bronchoscope may advance beyond the distal end of the endotracheal tube, potentially causing further trauma to the tracheal mucosa. In each group, 20 patients were randomly selected using a computer-generated random number list to undergo fibreoptic examination of the tracheal mucosa. This random selection was performed to ensure the subsample was representative of the overall cohort.

The bronchofiberscope was inserted via the tracheal tube until it reached the tracheal tube cuff. Then, the cuff was deflated, and the tracheal tube was extubated carefully to avoid the tip of the tracheal tube beyond the glottis. And the mucous where the tracheal tube cuff is located can evaluate directly by bronchofiberscope. Then, the tracheal tube was intubated to the original depth along the bronchofiberscope.

### Intervention procedure

The patients were enrolled one day before the operation and randomly divided into groups at a 1:1:1 ratio using a computer-generated random number table by the designer. The researcher, who supervised procedures such as intubation and cuff monitoring, had access to the opaque envelopes with the allocation numbers.Two separate assessors examined the outcome measures after surgery.The patients, outcome assessors, the statistician, and data collectors were all blinded to the allocation information. The resident physician responsible for assessing sore throat, hoarseness, cough, and mucosal injury had no access to the randomization list and was not present during cuff management. Endotracheal intubation was performed by an anaesthesiologist with more than five years of clinical experience.

The ETTc in the digital palpation method group (group A) was inflated by the anaesthesiologist according to his/her personal experience using the pilot balloon palpation method without any assistance of instrumentation. In the minimal occlusive volume method group (group B), the ETTc pressure was inflated with air until no exhalation sounds or leaks were heard by stethoscopic auscultation placed on the suprasternal fossa. Group A and group B will not make any further adjustments to the pressure after the inflation is completed. In the pressure control method group (group C), the ETTc was inflated first by the anaesthesiologist and then adjusted once by the researcher with a handheld aneroid manometer (VBM Medizintechnik, Sulz am Neckar, Germany) within the range of 25 to 30 cmH_2_O^10^. Air leakage around the ETT was monitored with a stethoscope. The cuff pressure was monitored continuously and maintained within the range of 25 to 30 cmH_2_O with a manometer throughout surgery in group C.

The primary outcomes were the incidence and severity of postoperative sore throat (POST). The incidence and severity of postoperative hoarseness, cough as secondary outcomes, The duration of the operation and endotracheal intubation were recorded in both groups. The ETTc pressure and airway pressure after intubation and mechanical ventilation (T_0_) were measured in three groups, and the ETTc pressure, airway pressure and pneumoperitoneum pressure were measured at 5 min (T_1_), 20 min (T_2_), 40 min (T_3_), and 60 min (T_4_) after insufflation. A resident physician was assigned to follow up the patients with a structured questionnaire and record endotracheal intubation-related airway complications, including sore throat, hoarseness, cough, and blood-streaked expectoration at 1 h, 6 h and 24 h after extubation. The severity of sore throat, hoarseness, and cough was graded using a 4-point scale (0: no, 1: minimal, 2: moderate, 3: severe) as follows. Sore throat: 0 = none, 1 = less severe than with a cold, 2 = similar to that noted with a cold, 3 = more severe than with a cold.^28^ Hoarseness: 0 = none, 1 = noted by the patient, 2 = obvious to the observer, 3 = aphonia. Cough: 0 = no cough, 1 = single cough, 2 = more than one episode of unsustained cough, 3 = severe sustained bouts of cough.^29^ The tracheal mucous injury was graded by self-made classification accordingly: 0 = indicates no injury, 1 = indicates punctate hemorrhage, 2 = indicates splinter hemorrhage, 3 = indicates ulcer.

### Statistical analysis

Based on preliminary data from our pilot study, the incidence of POST at 1 h after surgery in Groups A, B, and Group C was 48%, 26%, and 15%, respectively. Sample size estimation was performed using PASS 15.0 software (NCSS, LLC). A sample size of 141 achieves 90% power to detect an effect size (W) of 0.3003 using a 2 degrees of freedom Chi-Square Test with a significance level (alpha) of 0.05. Allowing for a dropout rate of 20%, a total of 180 patients were required, with an average of 60 patients in each group.

All relevant data from the included systems for each patient were combined into a single patient database. Data were processed and analysed with the SPSS 20.0 statistical software package, and *P* < 0.05 was considered statistically significant. Normally distributed measurement data were expressed as mean and standard deviation (SD), while non-normally distributed data were expressed as median and interquartile range (IQR). Categorical data were reported as numbers and percentages.Normally distributed measurement data (e.g., age, BMI) were compared using one-way ANOVA, whereas non-normally distributed data (e.g., blood loss) and categorical data (ASA classification) were analyzed using the χ^2^ test. To reduce the impact of the outliers, we compared tracheal mucous injury using the Kruskal-Wallis test. Differences in the incidence and severity of POST, hoarseness, and cough were analysed using the χ^2^ test or Fisher exact test. Furthermore, Bonferroni correlation was used to account for further statistical comparisons between groups, and *P* < 0.0167 (0.05/3) was considered statistically significant.

## Supplementary Information

Below is the link to the electronic supplementary material.

Supplementary Material 1

Supplementary Material 2

## Data Availability

The data associated with the paper are not publicly available but are available from the corresponding author on reasonable request.
