# Supplementary material for: Effects of different endotracheal tube cuff management on sore throat, hoarseness, and cough after short-term gynecological laparoscopic surgery
Source: Sci Rep. 2026 Apr 4;16:18997. doi: 10.1038/s41598-026-47172-8 (PMC13275885; doi:10.1038/s41598-026-47172-8)
Supplement: Supplementary file 2 — Supplementary Material 2 [file 41598_2026_47172_MOESM2_ESM.docx]

**Research protocol**

**Part1**

### **Project summary**

### Sore throat, hoarseness and cough are common airway complications after general anesthesia with tracheal intubation and can adversely affect patient satisfaction. In this study, we compared the effects of three different management for endotracheal tube cuff on postprocedural airway complications.

**Methods:** 180 patients undergoing gynaecological laparoscopic surgery (>60min or <180min) with general anesthesia were randomly divided into three groups(n=60, per group):digital palpation method group(group A), minimal occlusive volume method group(group B) and pressure control method group(group C). The tracheal mucosa of twenty patients in each group was observed by fiberoptic bronchoscopy until the endotracheal tube was removed.The incidence and severity of sore throat, hoarseness, and cough were measured at 1, 6, and 24 h after tracheal extubation.

**Discussion**: The study results will provide insight into the effects of three different management for endotracheal tube cuff on postprocedural airway complications, and may contribute to identify the best management of tracheal tube cuff protocol.

### **General information**

### The research site :Affiliated Hospital of North Sichuan Medical College

Qianli (Deputy chief physician): Conceptualization, Methodology, Formal analysis, Writing -Original Draft

Faping Tu (Resident doctor): Conceptualization, Methodology, Writing - Review & Editing

Xiaoling Jin( Resident doctor): Investigation, Data Curation

Ailing Tu(Chief physician): Data Curation

### **Rationale & background information**

The secure airway is of vital importance in patients with general anesthesia, which is often maintained by an endotracheal tube (ETT). Postoperative sore throat (POST), cough, and hoarseness are common airway complications after tracheal intubation, with an incidence varying from 14.4%-50% ^[1,2]^ The physiopathology of post-intubation airway symptoms is not completely clarified, but mucosal damage due to high cuff pressure is thought to be an essential causative factor ^[4-6]^. If the ETT cuff (ETTc) pressure is greater than 30 cmH_2_O, the airway mucosa could be damaged, and some airway complications would occur, in addition, the injury rate increases over time^[7]^.

Laparoscopic gynaecological surgery has become popular over the last decade, decreasing postoperative pain, shortening hospital stays, and reducing medical costs ^[8]^ . However, the CO_2_ pneumoperitoneum and Trendelenburg position during laparoscopy may increase ETTc pressure and lead to discomfort and complications postoperatively ^[9,10]^. Previous studies have shown that female patients are at a greater risk for POST after ETT ^[11-13)^.

Multiple randomized controlled trials suggested that during the long- during operation, continuous monitoring of the pressure of the tracheal tube and timely adjustment are necessary^[14,15]^. For surgery lasting only a few hours, most clinicians give little attention to inflation pressure of the ETTc and simply determine the pressure by pilot balloon palpation according to their experience.

### **Study goals and objectives**

The aim of the current study was to evaluate airway complications by measuring and controlling ETTc pressure in patients undergoing short-during laparoscopic gynaecological surgery with general anesthesia.

### **Study design**

This was a prospective, randomized, observational study. Written informed consent was obtained from all participants.

One hundred eighty patients aged between 18 and 60 **y**ears with American Society of Anesthesiologists (ASA) physical status I-II requiring elective laparoscopic gynaecological surgery that was expected to be no more than 3 h under general anesthesia with endotracheal intubation were prospectively investigated.

The exclusion criteria consisted of a history of recent respiratory tract infection, previous sore throat, hoarseness and cough, cigarette smoking, prior medication with analgesics or corticosteroids, history of heart or lung disease, previous neck surgery, Mallampati score 3 or 4, difficult intubation and decline in oxygen saturation to less than 95% during induction due to dif­ficult intubation, duration of tracheal intubation < 60 min or > 180 min, and use of a patient-controlled analgesia. The expected duration of the study was one year.

### **Methodology**

On the day of surgery, all participants were allocated into three parallel groups using an online randomization application designed for clinical research projects. Randomization was performed in balanced blocks with a 1:1:1 allocation ratio. The randomization list was generated using SAS Software version 9.4.

The researcher was not blinded to the group assignment; however, all of the other participants, including anaesthesiologists and follow-up resident physicians, were unaware of the assignment.

After endotracheal intubation, mechanical ventilation was provided by connecting the tube to the anesthesia machine (Fabius-Plus, Drager, Germany). The ETTc in the digital palpation method group (group A) was inflated by the anaesthesiologist according to his/her personal experience using the pilot balloon palpation method without any assistance of instrumentation. In the minimal occlusive volume method group (group B), the ETTc pressure was inflated with air until no exhalation sounds or leaks were heard by stethoscopic auscultation placed on the suprasternal fossa. In the pressure control method group (group C), the ETTc was inflated first by the anaesthesiologist and then adjusted once by the researcher with a handheld aneroid manometer (VBM Medizintechnik, Sulz am Neckar, Germany) within the range of 25 to 30 cm H_2_O. Air leakage around the ETT was monitored with a stethoscope. The ETTc pressure was measured soon after initial inflation of the ETTc and then maintained within the range of 25 to 30 cm H_2_O by the manometer in group C.

The duration of the operation and endotracheal intubation were recorded in both groups. The ETTc pressure and airway pressure after intubation and mechanical ventilation (T_0_) were measured in three groups, and the ETTc pressure, airway pressure and pneumoperitoneum pressure were measured at 5 min (T_1_), 20 min (T_2_), 40 min (T_3_), and 60 min (T_4_) after insufflation. A resident physician was assigned to follow up the patients with a structured questionnaire and record endotracheal intubation-related airway complications, including sore throat, hoarseness, cough, and blood-streaked expectoration at 1 h , 6 h and 24 h after extubation. The severity of sore throat, hoarseness, and cough was graded using a 4-point scale(0: no, 1: minimal, 2: moderate, 3: severe) as follows. Sore throat：0=none, 1=less severe than with a cold, 2=similar to that noted with a cold, 3=more severe than with a cold^[16]^. Hoarseness: 0=none, 1=noted by the patient, 2=obvious to the observer, 3=aphonia. Cough: 0=no cough, 1=single cough, 2=more than one episode of unsustained cough, 3=severe sustained bouts of cough^[17]^. The tracheal mucous injury was graded by self-made classification accordingly: 0=indicates no injury, 1=indicates punctate hemorrhage, 2= indicates splinter hemorrhage, 3= indicates ulcer.

At the end of the surgery, we utilized a fiberoptic bronchoscope (STERRAD 2227552,Olympus,Japan) to examine the tracheal mucosa.

### **Safety considerations**

The staff members conducting the clinical study must promptly act to ensure participant safety in the event of significant adverse occurrences throughout the trial and inform the management department and superiors.The research institution will ensure compliance with all legal and regulatory obligations through the reporting procedures.

All instances of unresolved adverse events (including, but not limited to, adverse responses) at the conclusion of the treatment program must be carefully monitored until a satisfactory resolution is attained or the situation stabilizes.

### **Follow-up**

A resident physician was assigned to follow up the patients with a structured questionnaire and record endotracheal intubation-related airway complications, including sore throat, hoarseness, cough, and blood-streaked expectoration at 1 h , 6 h and 24 h after extubation. The severity of sore throat, hoarseness, and cough was graded using a 4-point scale(0: no, 1: minimal, 2: moderate, 3: severe) as follows. Sore throat：0=none, 1=less severe than with a cold, 2=similar to that noted with a cold, 3=more severe than with a cold^(17)^. Hoarseness: 0=none, 1=noted by the patient, 2=obvious to the observer, 3=aphonia. Cough: 0=no cough, 1=single cough, 2=more than one episode of unsustained cough, 3=severe sustained bouts of cough^(18)^. The tracheal mucous injury was graded by self-made classification accordingly: 0=indicates no injury, 1=indicates punctate hemorrhage, 2= indicates splinter hemorrhage, 3= indicates ulcer.

### **Data management and statistical analysis**

The envelope containing the random number and grouping scenarios is entrusted to the principal experimenter for safekeeping. The "Case Report Form" must be completed in all instances, irrespective of whether the observation is finalized or withdrawn.

When the following emergency scenarios arise: Serious adverse events: The researcher notifies the primary person in charge and makes the decision to open the emergency letter if the individual requires emergency rescue. The case is not included in the statistical analysis once it has been opened and is considered a dropped case.

Upon completion of the research, the blinded data will be unblinded by a specialized research assistant. The relevant subject numbers are to be revealed to the individual accountable for data analysis. This procedure is subsequently reiterated for the conclusive analysis and findings of the investigation.

All relevant data from the included systems for each patient were combined into a single patient database. Data were processed and analysed with the SPSS 22.0 statistical software package, and *P*< 0.05 was considered statistically significant. Categorical variables were defined as numbers and percentages, and continuous variables are expressed as the mean ± standard deviation [SD]. For comparison of demographic data and anaesthetic consumption, Student’s t-test or Mann–Whitney U test was used as appropriate. To reduce the impact of the outliers, we compared the severity of POST, hoarseness, cough and tracheal mucous injury using the Kruskal-Wallis test. Differences in the occurrence of POST, hoarseness, and cough were analysed using Fischer’s exact test and Pearson Chi square test. Furthermore, Bonferroni correlation was used to account for multiple measurements within groups, and *P* < 0.0167 (0.05/3) was considered statistically significant.

### **Quality assurance**

Investigators must have professional expertise, qualifications and competence in clinical trials, which are determined after qualification screening, and the personnel requirements are relatively fixed.

Through preclinical trial training, the researchers gain a thorough comprehension of the clinical trial procedure and the unique meaning of each index. testing for consistency in the quantitative standards for indications and symptoms. Sign the statement provided by the investigator. Self-perceived symptoms should be described objectively, without being induced or prompted; the protocol's techniques and time points should be followed while examining the designated objective indicators. Adverse reactions or unanticipated toxicities should be observed and followed up.

Throughout the clinical study, make sure that the subjects' rights and interests are protected, that the data collected and reported is correct, comprehensive, and free of errors, and that the trial is conducted in accordance with the established protocol.

The results of this study will provide insights into the effect of PRP as adjuvant therapy to tendon fenestration and may help identifying the best preceding and concomitant rehabilitation protocol

### **Expected outcomes of the study**

The results of this study will provide insights into the effect of repeat intraoperative measurement of the endotracheal ETTc pressure on airway complications .And this is a simple and inexpensive procedure and should be used even in patients receiving short-duration laparoscopic surgery.

### **Duration of the project**

July 2016 to January 2017: application for ethical approval and literature search, pre-preparation of experiments

January 2017 to March 2018: completion of case collection

By 31 May 2018: completion of trial summary

By 31 August 2018: completion of statistical analysis

By 30 December 2019: completion of article

The whole subject time was about July 2016 to December 2019

### **Problems anticipated**

Patients do not cooperate with follow-up visits after surgery due to physical pain and other problems.

Solution: patient communication and full trust with the patient before and after surgery.

### **Ethics**

### Patients were picked during the preoperative visit to determine whether they met the inclusion requirements. If they did, they were introduced about the trial, its advantages and disadvantages, and asked if they wanted to participate in part. They were also told that they could withdraw from the study at any time and that the findings would be published without revealing any personal information. They sign an informed consent form if they approve.

### **Informed consent forms**

Signed (an omission)

**References**

1. Agarwal, A. et al. An evaluation of the efficacy of aspirin and benzydamine hydrochloride gargle for attenuating postoperative sore throat: a prospective, randomized, single-blind study. *Anesth. Analg.* **103**, 1001–1003; 10.1213/01.ane.0000231637.28427.00 (2006).
2. Jaensson, M., Gupta, A. & Nilsson, U. G. Risk factors for development of postoperative sore throat and hoarseness after endotracheal intubation in women: a secondary analysis. *AANA J*. **80**, S67–S73 (2012).
3. Macario, A., Weinger, M., Carney, S. & Kim, A. Which clinical anesthesia outcomes are important to avoid? The perspective of patients. *Anesth. Analg.* **89**, 652–658; 10.1097/00000539-199909000-00022 (1999).
4. Combes, X. et al. Intracuff pressure and tracheal morbidity: influence of filling with saline during nitrous oxide anesthesia. *Anesthesiology* **95**, 1120–1124; 10.1097/00000542-200111000-00015 (2001).
5. Svenson, J. E., Lindsay, M. B. & O'Connor, J. E. Endotracheal intracuff pressures in the ED and prehospital setting: Is there a problem? *Am. J. Emerg. Med.* **25**, 53–56; 10.1016/j.ajem.2006.09.001 (2007).
6. Seegobin, R. D. & Van Hasselt, G. L. Endotracheal cuff pressure and tracheal mucosal blood flow: endoscopic study of effects of four large volume cuffs. *Br. Med. J.* **288**, 965–968; 10.1136/bmj.288.6422.965 (1984).
7. Bernon, J. K. et al. Endotracheal tube cuff pressures - the worrying reality: a comparative audit of intra-operative versus emergency intubations. *S. Afr. Med. J.* **103**, 641–643; 10.7196/samj.6638 (2013).
8. Gerges, F. J., Kanazi, G. E. & Jabbour-Khoury, S. I. Anesthesia for laparoscopy: a review. *J. Clin. Anesth.* **18**, 67–78; 10.1016/j.jclinane.2005.01.013 (2006).
9. Yildirim, Z. B. et al. Changes in cuff pressure of endotracheal tube during laparoscopic and open abdominal surgery. *Surg. Endosc.* **26**, 398–401; 10.1007/s00464-011-1886-8 (2012).
10. Wu, C. Y. et al. Changes in endotracheal tube cuff pressure during laparoscopic surgery in head-up or head-down position. *BMC Anesthesiol.* **14**, 75; 10.1186/1471-2253-14-75 (2014).
11. Biro, P., Seifert, B. & Pasch, T. Complaints of sore throat after tracheal intubation: prospective evaluation. *Eur. J. Anaesthesiol.* **22**, 307–311; 10.1017/s0265021505000529 (2005).
12. Chen, K. T. et al. Risk factors associated with postoperative sore throat after tracheal intubation: an evaluation in the postanesthetic recovery room. *Acta Anaesthesiol. Taiwan* **42**, 3–8 (2004).
13. Ahmed, A., Abbasi, S., Ghafoor, A. H. & Ishaq, M. Postoperative sore throat after elective surgical procedures. *J. Ayub Med. Coll. Abbottabad* **19**, 12–14 (2007).
14. Puthenveettil, N. et al. Effect of cuff pressures on postoperative sore throat in gynecologic laparoscopic surgery: an observational study. *Anesth. Essays Res.* **12**, 484–488; 10.4103/aer.AER_72_18 (2018).
15. Cai, S. et al. Changes of endotracheal tube cuff pressure and its indicators in laparoscopic resection of colorectal neoplasms: an observational prospective clinical trial. *BMC Anesthesiol.* **24**, 413; 10.1186/s12871-024-02802-4 (2024).
16. El-Boghdadly, K., Bailey, C. R. & Wiles, M. D. Postoperative sore throat: a systematic review. *Anaesthesia* **71**, 706–717; 10.1111/anae.13438 (2016).
17. Harding, C. J. & McVey, F. K. Interview method affects incidence of postoperative sore throat. *Anaesthesia* **42**, 1104–1107; 10.1111/j.1365-2044.1987.tb05179.x (1987).
18. Minogue, S. C., Ralph, J. & Lampa, M. J. Laryngotracheal topicalization with lidocaine before intubation decreases the incidence of coughing on emergence from general anesthesia. *Anesth. Analg.* **99**, 1253–1257; 10.1213/01.ANE.0000132779.27085.52 (2004).

**Part2**

### **Other support for the project**

This work did not have any financial support.

### **Collaboration with other scientists or research institutions**

No.
